# Chicago Medical Society

**Published:** 1879-04

**Authors:** R. Park


### ARTICLE X.

CHICAGO MEDICAL SOCIETY. Reported by R. PARK, M.D. Meeting, March 17th. Dr. Ingals in the chair.

Prof. E. L. Holmes read a paper on "Atropine, Duboisine, Eserine, Pilocarpine and Muscarine," which will be found under the original communications of this number.

In the discussion which followed, Dr. F. C. Hotz remarked that since the reading of his paper on anomalous effects of atropine, he had observed several cases in which the intra-ocular tension was greatly diminished by the prolonged use of atropine. In one case the eye was exceedingly sensitive to atropine, but tolerated the drug after morphine was added to it. It was a case of acute iritis of the right eye of a woman, aged thirty-one years, who consulted the doctor in the middle of December. The inflammation had lasted three weeks, and consequently there were several posterior synechiæ; the iris was much discolored, a very intense peri-corneal injection, and great pain in and about the eye; V=fingers across the room, tension normal. After one week the patient reported that the two preceding days the atropine had caused her much pain; and the eye which had been improving at first, was again more inflamed, the pupil more contracted. Morphine was added to the solution of atropine, and under this medicine the eye rapidly recovered, so that a few days after New Year's it was quite well and had perfect sight. But, at the end of January, patient returned with the eye worse than ever. It was very red and painful, but the pupil was of medium size, and regular. The sight, however, was again reduced to the counting of fingers across the room, and the tension of the globe was exceedingly diminished, and it was as soft as a partially atrophied eyeball. Patient had been using atropine all through January. It was discontinued at once; a mere placebo was given (an ointment with vaseline to rub in the forehead) and after one week the eye was well and its sight perfectly restored.

In regard to pilocarpine and eserine, Dr. H. thought that pilocarpine would probably be the myotic of the future, because it is more stable in solution than eserine; furthermore, it produces less irritation, and probably it will be less expensive. "Both remedies are still on trial, and nothing definite can be said about the indications for their use. But if some writers recommend them in cases of hypopyon, it is difficult to harmonize their advice with the facts that all observers consider pilocarpine or eserine strictly contra-indicated in affections of the iris, and that in hypopyon the iris is almost always implicated."

Pilocarpine and eserine are considered very efficient in reducing the tension of the eye-ball. But, in the following case, pilocarpine seemed to have an opposite effect. A sailor came to the Illinois Eye and Ear Infirmary a few weeks ago with a large central ulcer of the right cornea. It came on three months ago, after an eruption of herpes zoster frontalis and nasalis. Atropine has been used all the time ; pupil was moderately dilated, eye-ball very red, painful and extremely soft. Pilocarpine was used in lieu of atropine with a very good result; the pain has left the eye, the inflammatory symptoms have pretty nearly disappeared, and the tension of the globe has decidedly improved, though it is still a little under the normal standard.

Dr. Lyman Ware wanted to ask whether the use of pilocarpine would do away with the necessity for iridectomy in cases of glaucoma? He had been treating a sub-acute case of this disease with it, but sufficient length of time had not elapsed to prove its efficacy.

Dr. Holmes closed the discussion by saying that the only glaucomatous case in which he had used the drug was one complicated by staphylomatous cornea, and, although it seemed to work well, he was not yet willing to commit himself on this point. In acute cases he thought that very few would be willing to wait long enough to test it, fearing disastrous result from the delay.

He apprehended danger in the future from the use of these drugs in unskillful hands, since very few were able to diagnose iritis in its earliest incipiency, and if an inflamed iris should be thus contracted down by their use, adhesions might form and irreparable injury be the result.

Dr. R. G. Bogue then reported a case of intra-laryngeal tumor in the left aryteno-epiglottic fold occluding the glottic opening, the case being complicated by a chronic pharyngitis and ulcerated epiglottis. After the use of local astringents and general tonics, he performed tracheotomy, leaving the tube *in situ* some nine months. During this time and under the internal use of potassic bromide, the tumor became smaller, and now, after three years, only a scar remains to mark its site. In discussing its nature he said that if it were considered of a specific character, of which there were no constitutional evidences, it would indicate that the bromide was as efficacious as the iodide was reputed to be.

Dr. Bogue also reported the case of a woman aged 24 years, in the puerperal state, who was suddenly seized with acute pain in the calf of one leg. After a few days this was followed by discoloration of the foot, and this by spontaneous gangrene. In spite of all treatment this extended, and finally, a line of demarcation having formed, he amputated through the knee joint successfully. Subsequent dissection of the amputated portion revealed extensive occlusion of the venous channels from a phlegmasia of the *deep* veins, which was a very rare occurence. The patient's recovery was delayed by a mild phlegmasia alba dolens of the other leg. He exhibited sections of the occluded popliteal veins, of which there were two.

Dr. D. W. Graham exhibited a femur, removed from a subject supplied for his lecture course, which gave evidence of an oblique fracture having at some time been suffered involving the upper third of the shaft; the upper fragment having been tilted forwards and outwards. The bone, on comparison with its fellow showed an inch of shortening, one-third of which was between the two trochanters, thus indicating that the lesser trochanter formed a part of the lower fragment. The peculiar obliquity and locality of the fracture made the specimen an interesting and unique one.

Dr. Roswell Park exhibited a specimen, also removed from a dissecting room subject, of an elbow joint which had been the seat of some injury which he conceived to have been of the following nature: An oblique fracture of the upper one-fourth of the shaft of the ulna, with dislocation forwards of the head of the radius. The result of the injury was deformity of the ulna near its olecranon process from gliding and rotation of the fragments, and a new socket for the head of the radius on the external condyle of the humerus, an inch above its proper site on the ulna, where it had formed strong ligamentous attachments. The head of the bone was much larger than normal, evidently from irritation, and those parts of the condyle and of the radial head which were in contact, were eburnated and denuded. In the outer part of the orbicular ligament was what seemed to be a calcified fragment of periosteum, which had probably been loosened at the time of the injury.

After inspection, all those present agreed in accepting the above explanation as best accounting for the appearances of the specimen.

The society then endorsed a resolution passed by the West Chicago Society recommending the State Board of Health to secure legislation providing for compensation of persons who make returns of births and deaths, as now required by law; after which it adjourned.

---

RESEARCHES ON THE TREE WHICH PRODUCES "GOA" POWDER.—Dr. Da Silva Lima. "The tree which yields the Araroba or Goa powder is known in the districts where this industry flourishes under the name *Angelim amargosa* ('bitter angelim'). The word *angelim* is not now understood. The tree belongs to the nat. ord. Leguminosæ, and the appellation 'bitter' arises from the fact that the ligneous portion resembles good cinchona in flavor and bitterness. It is found in company with another tree belonging probably to the same genus, namely, *Andira anthelmintica* Benth., which has anthelmintic properties. There is also an *angelim doce* ('sweet angelim,' *Andira vermifuga*) and an *Angelim pedra* (*Andira spectabilis*)."

The araroba tree occurs abundantly in the forests of Camamu, Igrapiuna, Santarem, Taperoa and Valença, of the province of Bahia. It attains a very large size, one to two meters in diameter, and twenty to thirty meters in height.

The Goa powder is contained in more or less narrow fissures and chinks in the ligneous portion, running mostly through the whole length of the trunk, and becoming narrower above. It is customary to cut down the tree, to saw it into sections, and then to split the blocks open in the direction of the fissures, when the powder is readily obtained. There is scarcely any doubt that the original tree is either an *Andira* or a *Cæsalpinia*.—*L'Union Pharmaceutique.*

